# The Influence of a Single Instrument-Assisted Manual Therapy (IAMT) for the Lower Back on the Structural and Functional Properties of the Dorsal Myofascial Chain in Female Soccer Players: A Randomised, Placebo-Controlled Trial

**DOI:** 10.3390/jcm11237110

**Published:** 2022-11-30

**Authors:** Patrick Weber, Werner Klingler, Robert Schleip, Nadine Weber, Christine Joisten

**Affiliations:** 1 Department for Physical Activity in Public Health, Institute of Movement and Neurosciences, German Sport University Cologne, 50933 Cologne, Germany; 2 PANOVIA Medical Cooperative Society, 50354 Hürth, Germany; 3 Anaesthesiology, SRH Hospitals Sigmaringen, 72488 Sigmaringen, Germany; 4 Experimental Anaesthesiology, Ulm University, 89081 Ulm, Germany; 5 Clinical Sciences, Queensland University of Technology, Brisbane, QLD 4000, Australia; 6 Conservative and Rehabilitative Orthopaedics, Department of Sport and Health Sciences, Technical University of Munich, 80809 Munich, Germany; 7 Department for Medical Professions, Diploma University of Applied Sciences, 37242 Bad Sooden-Allendorf, Germany

**Keywords:** instrument-assisted, myofascial, placebo-controlled, lumbar fascia, athletes, ultrasonography, connective tissue, manual therapy, treatment, range of motion

## Abstract

Background: Instrument-assisted manual therapy (IAMT) is indicated to improve flexibility, reduce pain, and induce hyperaemia locally and along myofascial chains. The underlying effects are largely unclear. This randomised, placebo-controlled pilot study aimed to gain first insights into these effects, primarily on the structural level, through ultrasonography. Methods: 67 healthy female soccer players aged 20.9 (±3.9) years were examined after right lumbar intervention (IAMT: intervention group (IG), heat application: comparison group (CG), pressure-less placebo: placebo group (PG)). Ultrasonography (absolute movement and shear motion), flexibility tests (passive straight leg raise test (PSLR), lumbar and thoracic double inclinometry), and superficial skin temperature were recorded before (t0), immediately (t1) and 45 min after the intervention (t2). Results: IAMT decreased the absolute mobility of the superficial lamina and its shear motion to the superficial fascia compared with the PG (t1; *p* < 0.05). PSLR improved in the IG compared with the CG (t2) and PG (t1, t2; *p* < 0.05). The temperature increased in the IG and CG compared with the PG (t1, t2) and in the CG compared with the IG (t1; *p* < 0.05). Conclusion: IAMT of the lumbar back briefly reduces absolute mobility of the superficial lamina and its shear motion to the superficial fascia, improves flexibility, and increases the temperature.

## 1. Introduction

Instrument-assisted manual therapy (IAMT) is increasingly applied for the rehabilitation of soft tissues [1] and the enhancement of movement dynamics [2,3,4]. In these contexts, the primary goal is to generate pressure, tensile, and shear forces [5,6] to elicit targeted therapeutic deformation of specific fibrous tissues, large tissue areas, and deep tissue layers [4,5,6,7,8]. As a result of mechanical stimulation, IAMT is reported to provide pain relief [2,5,6,9,10], locally increase blood flow with an accompanying increase in superficial skin temperature [3,6,9] and improve functionality and range of motion (ROM) with reduced joint stiffness [2,3,4,5,10,11,12]. The mechanisms of action and underlying effects are largely unclear. However, they are assumed to involve physiological adaptive reactions [10] such as increased microcirculation [3,6,9,13], activation of fibroblasts [14], and enrichment of cytokines in the ground substance, particularly growth factors and inflammatory mediators for regeneration and stimulation of wound healing [5]. A reduced joint stiffness is believed to improve ROM [11] owing to reduced muscle–tendon [15] or soft tissue stiffness surrounding the joint [11]. Similarly, myofascial release [3,8], reduced tissue viscosity [3], improved sliding properties between tissue layers [9], release of restrictions in the fascial and scar tissues [3], and inhibition of pain receptors [3,9] are hypothesised to contribute to the therapeutic effects. Additionally, the first postoperative intervention results showed that manual mobilisation prevents adhesions [16].

In athletes, IAMT aids in acute and chronic soft tissue injury recovery [14] and improves hamstring flexibility compared with static stretching [17]. Hamstring injuries are of primary concern, especially to soccer players, because they cause long absences from the sport [18,19]. The most important risk factors for these injuries are muscle tightness [20] and low hamstring flexibility [20,21]. Owing to the known mechanical relationship between the hamstrings and lower back, improvement in hamstring flexibility and reduction in lower back pain (LBP) in people with nonspecific LBP were achieved after applying IAMT only to the dorsal thigh [22]. In this context, the thoracolumbar fascia (TLF) as a mechanical pivot and an anchor point of the dorsal chain [23,24,25,26,27] and a main actor in the force transmission between the upper and lower bodies [23,25,26,27] plays an important role. Under physiological conditions, the myofascial connections are exposed to high peak loads of up to 160% of the original muscle force owing to the interaction with the synergists [28]; this can lead to visible changes in the fibrous tissue [29] and consequently to negative influences on the myofascial system [30,31] and musculoskeletal dynamics [32]. Structural alterations and a significantly reduced shear motion (SM) of the TLF have been demonstrated on ultrasound imaging in patients with chronic LBP [30,31]. Conversely, a significant improvement in the mobility of the TLF in healthy individuals was detected after a single foam roller intervention of the dorsal chain [33]. 

Despite the initial findings in the field of IAMT, the data, underlying effects, and evidence remain very weak [2,12,34,35]. Criticism focuses mainly on the quality of the methodological approach [2,12,14,34,35] and the lack of treatment protocol standardisation [34,35,36]. Consequently, further studies with sufficient methodological quality are needed to determine the underlying effects and mechanisms of action of IAMT.

To gain more precise insights into the effects of a single standardised IAMT on the structural and functional properties of the myofascial tissues of the lower back and adjacent movement segments in a homogeneous collective, we integrated healthy female soccer players into the present pilot study. We aimed to analyse structural movement outcomes, including the absolute movement and SM of different tissue layers (primary outcome), and functional movement outcomes, including the flexibility of the hamstrings and ROM of the lumbar and thoracic spines (secondary outcome).

IAMT of the lower back was hypothesised to yield an inverse interaction with improved hamstring flexibility and an increased sliding mobility of the tissue layers. Furthermore, an increase in the superficial skin temperature and an improvement in the lumbar and thoracic ROM were expected.

## 2. Materials and Methods

### 2.1. Study Design

The study design used had been published by Weber et al. (2020) and was pre-registered at the German Clinical Trials Register (DRKS00012252) on 20 June 2018 [37]. The initial results of the published study design for the functional and structural movement parameters in combination with the superficial skin temperature were presented to make an initial statement about the effects of IAMT on the functional and structural movement properties of the dorsal chain.

Herein, athletes were randomised into three even groups (1:1:1) via an urn (adaptive biased coin) design, using printed cards in closed envelopes indicating group allocation [37,38,39]. The intervention group (IG) received a standardised IAMT. The comparison group (CG) underwent heat therapy with an applied heat source, while the placebo group (PG) received a pressure-less placebo treatment once on the right (R) side of the lower back. The CG was utilised to calculate the intervention effects based on the locally increased blood flow and microcirculation attributed to IAMT [3,6,9,13] as well as heat application [13,40,41,42]. All treatments were performed by the same therapist, while all outcome measures were collected using a standardised protocol by a blinded investigator. Measurements were performed immediately before (t0), immediately after (t1), and 45 min after the treatment (t2), at which time the heat application effects should have already subsided [40,42]. Blinded analyses were conducted a few weeks after data collection.

### 2.2. Participants

Sixty-seven healthy senior-level female soccer players who were actively involved in competition, practised at least thrice per week for 90 min each session, and were aged 15–35 years were recruited. The participants had not experienced back pathology within the past 4 weeks and had no chronic illnesses or acute injuries. After receiving detailed information about the study, all participants provided their consent. The study was approved by the ethics committee of the German Sport University Cologne (No. 80/2017) and conducted in accordance with the latest version of the Declaration of Helsinki.

Inclusion criteria

1. Female soccer player;
2. Age of 15–35 years;
3. Good health;
4. Active involvement in competition;
5. Minimum of three 90 min practice sessions per week.

Exclusion criteria

1. Back pathology within the past 4 weeks;
2. Chronic illnesses;
3. Acute injuries.

### 2.3. Measurements

**Figure 1:**
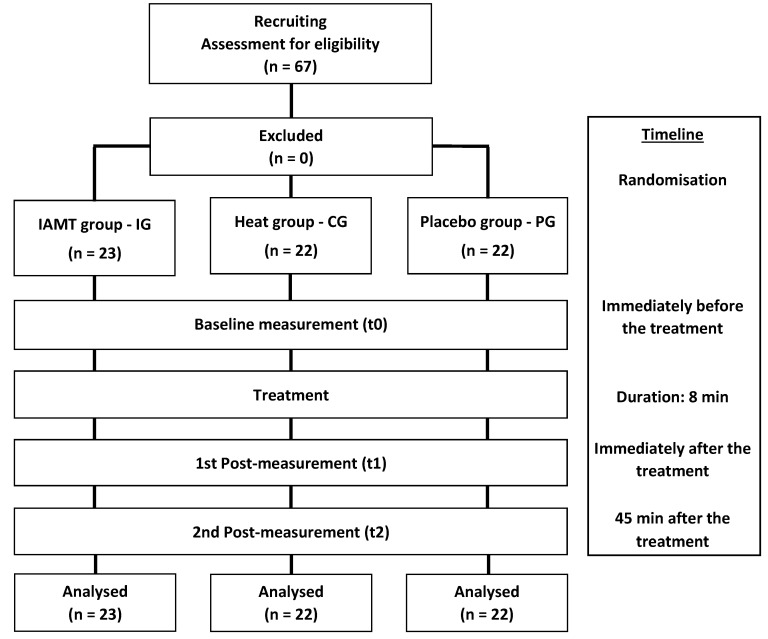
Schematic representation of the study design. IAMT: instrument-assisted manual therapy; IG: intervention group; CG: comparison group; PG: placebo group. Adapted from the original by Weber et al. (2020) [37].

Anthropometric data were obtained together with questionnaire data, including career, practice session, lifestyle, and subjectively perceived state of the lower back and flexibility. The athletes were undressed down to their underwear, and all measurement points were cleaned and marked. Thereafter, both body sides were tested alternately, according to the standardised measurement protocol presented by Weber et al. (2020) [37], starting with a superficial skin temperature assessment, followed by ultrasound measurements, and concluded with the flexibility tests, starting with the treatment side each time. For all measurements, except for flexibility, the participants lay prone with the anterior superior iliac spine positioned above the rotation axis of the Stockholm treatment table (Clap Tzu GmbH, Nordenham, Germany). The participant’s legs were stretched out, with their feet on a half-round soft roller (diameter: 10 cm), and their arms lay beside the upper body. After baseline measurement, each athlete received the treatments, followed by post-intervention measurements (Figure 1). All measurements were taken three times per time point, except for flexibility and ultrasound data. Mean values were calculated for comparative analysis and presented with their corresponding standard deviations (±SD). Room temperature was constant between 23.7 (±0.9) °C and 24.1 (±0.5) °C in all three groups throughout the study. Similarly, there were no differences in the required examination time (IG: 108.0 (±6.0) min, CG: 110 (±5.0) min, PG: 106.0 (±4.0) min).

#### 2.3.1. Ultrasound Imaging

**Figure 2:**
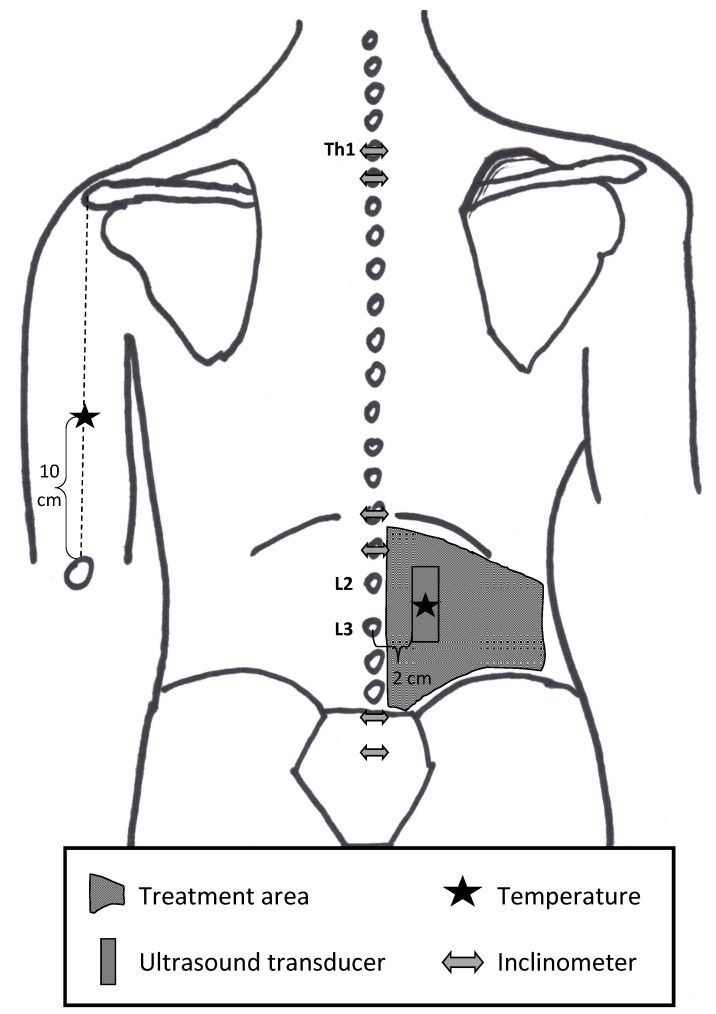
Body chart for schematic representation of the measurement points, shown on the treatment side. Th: thoracic vertebra; L: lumbar vertebra. Adapted from the original by Weber et al. (2020) [37].

The absolute movement and SM of the single tissue layers were investigated (SonidoSmart Plus, Zimmer MedizinSysteme, Neu-Ulm, Germany), while the medial edge of a 3.8 cm long linear transducer (16 MHz) was centred longitudinally 2 cm lateral to the spinous processes of lumbar vertebrae (L) 2 and L3 (Figure 2). A cine-loop technique with a single ultrasound beam at a depth of 3.2 cm focused on the posterior layer of the TLF was used while the treatment table was pivoted into a 45° roof position at a speed of 3° per second [30,33].

**Figure 3:**
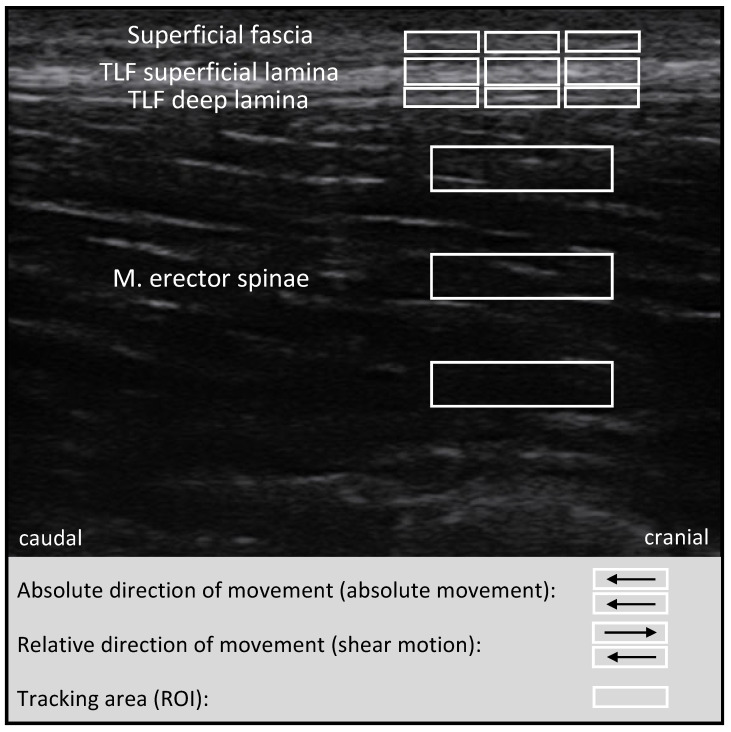
Schematic replica of Dilley’s (2001) shear motion analysis. TLF: thoracolumbar fascia; M.: muscle; ROI: region of interest. Adapted from the original by Weber et al. (2018) [53].

Motion analysis of the tissue layers was conducted using the cross-correlation software (CCS) Motion Analysis 2014v1 developed by Dilley et al. (2001) and used by Griefahn et al. (2017) to analyse the absolute mobility of the TLF in millimetres [33,43]. The CCS converted the videos created with the cine-loop technique into image sequences of 21 frames per second. Three regions of interest (ROI) were marked carefully on each of the following tissue layers without passing the layers’ borders to prevent analysis errors: superficial fascia (SF), superficial lamina (SL) of the posterior layer of the TLF, and deep lamina (DL) of the posterior layer of the TLF and erector spinae muscle (ESM). All ROI were set next to each other [33], except for those for the ESM, whose ROI were set one below the others to cover the entire depth at the middle of the right side of the image (Figure 3). The CCS analysed each pixel shift between two consecutive frames within each ROI via cross-correlation. It used the frame-by-frame results to calculate the horizontal tissue motion during the passively induced lumbar flexion in millimetres [43]. The average of the three ROI was used to determine the absolute motion of each tissue layer, which was analysed separately. All analyses were checked manually, and incorrectly tracked ROI were removed from the averaging. Finally, the relative motion between two adjacent tissue layers, the SM, was defined as the difference between the lower and upper layers in millimetres (Figure 3), e.g., TLF−SL − SF = SM (SF/TLF−SL).

#### 2.3.2. Dorsal Structure Flexibility Tests

Hamstring flexibility was evaluated using the passive straight leg raise test (PSLR). The athletes rested in the supine position while the investigator moved the extended leg into maximum passive hip flexion and determined the ROM in degrees using an inclinometer (AcuAngle, Baseline, Elmsford, New York, NY, USA) [44,45] placed at the lateral malleolus.

Lumbar spinal flexibility was determined using double inclinometry. One inclinometer was placed over the motion segment of the thoracic vertebra (Th) 12 and L1 and the other at the sacral base in an upright position (Figure 2). The participants performed maximal spinal flexion with extended legs, initially eccentrically by yielding to gravity and then concentrically with active muscle engagement. The following instructions were provided to guide the participants:

1. Starting with your head, bend your upper body forward with your knees extended, and let gravity passively guide you towards the floor, so that your extended arms are as close to the floor as possible.
2. Now, try to reach the floor through muscular activation with your fingertips or palms while your knees remain fully extended.

The difference between the inclinometric data was used to determine flexibility in degrees [30,46,47,48]. The thoracic spine was tested using the same protocol. For these measurements, the sacral inclinometer was transferred to the spinous processes of Th1 and Th2, while the other inclinometer retained its position [49,50,51,52].

#### 2.3.3. Superficial Skin Temperature Assessment

The superficial skin temperature was determined in degrees Celsius using a multifunctional thermometer (FT70, Beurer Medical, Ulm, Germany) at the treatment area 2.5 cm lateral to the interspinous space of L2 and L3 and the reference point on the contralateral musculus triceps brachii (Figure 2).

### 2.4. Treatments

All treatments were performed in the prone position in the 45° roof position. The treatment area ranged from the iliac crest up to the 12th rib and from the spinous processes to a connecting line of the iliac crest and costal arch (Figure 2). All treatments lasted 8 min based on the intervention duration of the IAMT treatment.

#### 2.4.1. IAMT Treatment

**Figure 4:**
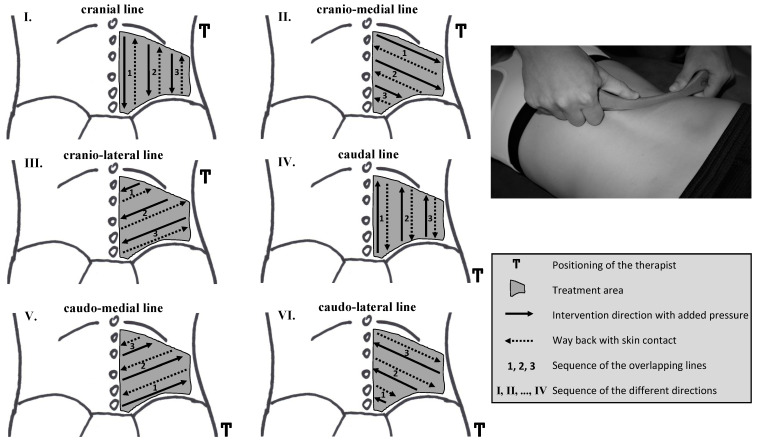
Treatment sequence of the instrument-assisted manual therapy techniques in chronological order. In the photograph, treatment is demonstrated on the cranio-lateral line using a Fazer 2 from ARTZT vitality (Ludwig Artzt GmbH, Dornburg, Germany). Adapted from the original by Weber et al. (2020) [37].

IAMT comprised two techniques and was applied using the Fazer 2 instrument (Ludwig Artzt GmbH, Dornburg, Germany). The first technique consisted of short, shock-like frictions called ’metabolisation’. For the second technique, called ’rehydration’, the Fazer tool was moved slowly through the tissue, pushing a shifting skin fold in front of the tool in the direction of movement. Both techniques were performed with the direction of movement away from the therapist (Figure 4), and the Fazer tool’s convex side was applied to reach but not exceed the pressure pain threshold defined by Rolke et al. (2010) [54]. To determine this point, the investigator instructed the athletes the following:

Please tell the therapist, through verbal feedback, when the treatment intensity reaches a maximum pressure sensation, but just before reaching a transition to a discomfort that causes aching, burning, drilling, or stinging sensations.

A lubricating pH-neutral cream was applied to the site of treatment. Next, the two techniques were conducted one after the other in six different directions with three overlapping lines to achieve the highest number of different fibre courses (Figure 4). After completing three lines in one direction, a second sequence was applied before continuing with the next direction and the other technique (Figure 4) [37].

After each treatment technique, the technique was reproduced thrice on a scale (DH-252G, Villeroy & Boch, Mettlach, Germany) to define the individual pressure applied to the athlete in kilogrammes. A small towel was placed between the Fazer tool and scale to improve gliding. The highest deflection per repetition was used to calculate the mean value. Video monitoring ensured the quality standards of the intervention and aided in determining the treatment speed of the two techniques. The applied force averaged 6.7 (±0.9) kg during metabolisation and 7.8 (±1.2) kg during rehydration. The mean treatment speed was 6.2 (±0.3) cm/s for metabolisation and 2.1 (±0.1) cm/s for rehydration.

#### 2.4.2. Heat Treatment

Heat treatment was applied using a 15 cm^2^ hot pack with an initial temperature of 55 (±2.5) °C, commonly used in clinical practice [42,55,56,57].

#### 2.4.3. Placebo Treatment

Placebo treatment was performed by moving an inactivated 5 cm^2^ ultrasound transducer at a moderate pace and applying a conductive gel in a circular manner without added pressure.

### 2.5. Statistical Analysis

The sample size was calculated using G*Power 3.1 (Heinrich Heine Universität Düsseldorf, Germany) [58]. Assuming a statistical power of 80% and accepting an alpha error of 0.05 with the expectation of a large effect size [33], we calculated a total required sample size of 64 athletes. Statistical analysis was performed using SPSS Statistics 27 (IBM, Armonk, New York, NY, USA). Ordinary descriptive statistics were used to summarise the results. Means for each group and mean differences were presented with SDs. The baseline sample characteristics of the groups and all main outcomes, e.g., the SM (distance in millimetres) and flexibility (ROM in degrees) in the three measurement time points, were compared using single-factor analysis of variance or the Kruskal–Wallis test in cases of non-normal data distribution. Qualitative variables, such as experience in myofascial self-treatment and ‘preferred free leg‘, were analysed using the chi-squared test. For three values collected, mean values were calculated for comparative analysis. Furthermore, delta (Δ1) values were calculated using the first post-intervention measurement (t1) and baseline measurement (t0) for a mean comparison immediately after the intervention. For a mean comparison 45 min after the intervention, delta (Δ2) values were calculated using the second post-intervention measurement (t2) and baseline measurement (t0). The significance level for all tests was set at *p* < 0.05.

## 3. Results

The soccer players’ mean age was 20.9 (±3.9) years; average height 167.4 (±5.5) cm; average weight 62.6 (±7.0) kg; and average body mass index 22.3 (±2.3) kg/m^2^. The players had an average of 12.4 (±4.2) years of sports experience and a regular weekly training volume of 7.2 (±2.0) h, corresponding to an average of 1.0 (±0.3) h per day. The three groups did not differ in any of these parameters. There were also no differences in the amount of training during the past 7 days or menstruation phase on the day of the examination, and subjectively perceived mobility and condition of the lower back at the beginning of the examination (Table 1). Similarly, the playing leg, performance level, squad membership, hormonal contraceptive use, or myofascial self-release exercise history did not significantly differ between the groups (Table 1).

### 3.1. Structural Movement Parameters

**Figure 5:**
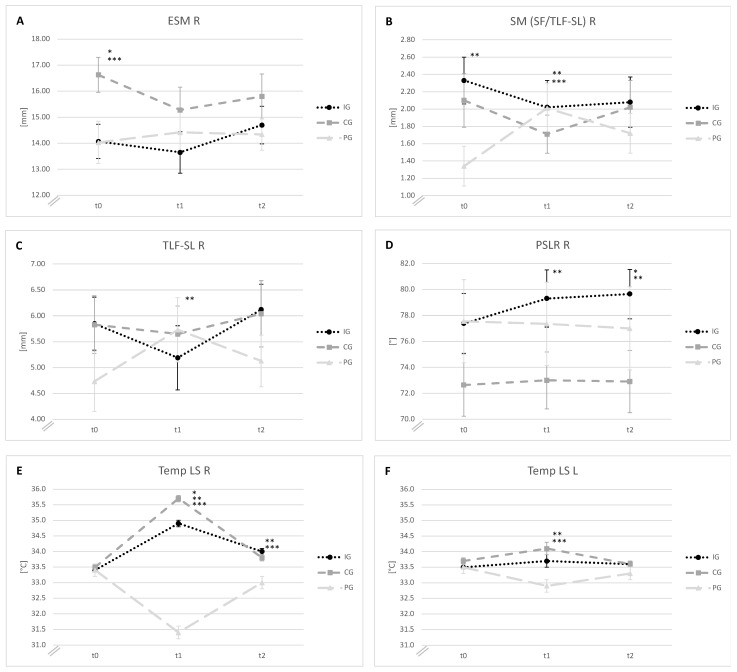
(**A**) Absolute movement of the paravertebral muscles on the right side of the lumbar spine (ESM R) in millimetres. (**B**) Shear motion between the superficial fascia and the superficial lamina of the thoracolumbar fascia on the right side (SM (SF/TLF-SL) R) in millimetres. (**C**) Absolute movement of the superficial lamina of the thoracolumbar fascia on the right side of the lumbar spine (TLF-SL R) in millimetres. (**D**) Passive straight leg raise test to determine hamstring flexibility on the intervention side (PSLR R) in degrees. (**E**) Superficial skin temperature of the intervention area (Temp LS R) in degrees Celsius. (**F**) Superficial skin temperature of the control side (Temp LS L) in degrees Celsius. The mean values ±SE at the three measurement time points (t0: before the intervention; t1: immediately after the intervention; t2: 45 min after the intervention) are shown. *p* < 0.05 * IG vs. CG, ** IG vs. PG, *** CG vs. PG. IG: intervention group; CG: comparison group; PG: placebo group.

At baseline, the absolute movement of the ESM on the right side showed a greater SM in the CG than in the other groups (*p* < 0.05) (Table 2 and Figure 5A). In addition, there was a greater SM between the SF and TLF-SL on the right side (SM (SF/TLF-SL) R) in the IG than in the PG (*p* < 0.05) (Table 2 and Figure 5B).

Immediately after the intervention, the TLF-SL showed reduced absolute mobility on the intervention side in the IG compared with that in the PG (*p* < 0.05) (Table 2 and Figure 5C). The SM (SF/TLF-SL) R also showed reduced gliding mobility in the IG and CG compared with that in the PG (*p* < 0.05) (Table 2 and Figure 5B). At t2, there was a difference in the between-group effect (*p* < 0.05) on the SM between the TLF-DL and the ESM on the left side. However, no difference was observed in the post hoc test (Table 2).

### 3.2. Functional Movement Parameters

The baseline functional movement parameters did not differ between the groups (Table 3). The PSLR on the intervention side showed an improved ROM in the IG compared with that in the PG immediately after the intervention (*p* < 0.05) (Table 3 and Figure 5D). Forty-five minutes after the intervention, the IG showed an improved ROM in the PSLR on the intervention side compared with the CG and PG (*p* < 0.05) (Table 3 and Figure 5D).

### 3.3. Superficial Skin Temperature

At baseline, the superficial skin temperature did not differ between the groups (Table 4). At t1, the temperature on the intervention side increased in the CG compared with that in the IG and PG (*p* < 0.05). The temperature on the intervention side also differed between the IG and PG (*p* < 0.05) (Table 4 and Figure 5E). In addition, the superficial skin temperature on the left lumbar side differed between the IG and PG and between the CG and PG (*p* < 0.05) (Table 4 and Figure 5F). The differences on the intervention side were maintained 45 min after the intervention between the IG and PG and between the CG and PG (*p* < 0.05) (Table 4 and Figure 5E). The temperature of the reference point showed no changes at any time point. In addition, a clear reddening of the skin was observed in the intervention area in the IG and CG immediately after the intervention and was still visible in the IG but completely disappeared in the CG 45 min after the intervention. The skin colour of the PG remained unchanged during the entire examination period.

### 3.4. Superficial Fascia Changes

**Figure 6:**
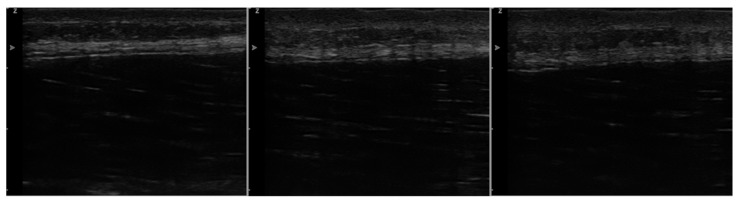
Observed changes in the area of the superficial fascia before instrument-assisted manual therapy (IAMT) intervention (**left**), immediately after IAMT intervention (**middle**), and 45 min after IAMT intervention (**right**) on the ultrasound images.

In the IG, the SF was swollen immediately and 45 min after IAMT on the ultrasound images (Figure 6). A ’fraying’ above and below the SF accompanied by a lowering of the fascial structures (internally) was also noted.

## 4. Discussion

To our knowledge, the present pilot study presents the first quantitative results demonstrating the effects of a single IAMT of the lumbar area on the structural and functional properties of the lower back and adjacent motion segments.

The most striking finding on the structural level was the significant decrease in the absolute movement of the TLF-SL immediately after IAMT compared with that after the placebo treatment. A conceivable explanation for this short-term effect is a change in the hyaluron-binding form into a higher molecular condition [59,60] and the associated increase in viscosity and decrease in tissue-specific lubricity [61,62,63]. Essentially, decreased viscosity along with increased ROM, flexibility, and tissue temperature is reported after manual manipulation [63], which is consistent with the results at t2 and supports the measured structural movement improvement. Another conceivable explanation is the withdrawal of blood volume from the superficial to deeper structures in response to the moderate cold stimulus [64] in the PG, which may cause hyperaemia and short-term warming of these tissue layers. Through the constant behaviour of the CG, it can be hypothesised that the TLF-SL reacts to a slight cold stimulus with a short-term improvement and to a vigorous mechanical stimulation with a short-term reduction in absolute movement. This hypothesis can be expanded to the non-significant observations in the SF, TLF-DL, and ESM, and additionally to the two shallow layers (SF and TLF-SL) of the control side after IAMT.

Apart from the influence of the hyaluron-binding form, mechanical stimulation of the myofascial tissue and the resulting pressure, tensile, and shear forces influence the structural conditions [5,6,33]. IAMT has been credited with improvements in functionality and ROM and reductions in joint stiffness [3,4,5,10,11]. Manual mobilisation is thought to sustainably reduce adhesions between the individual tissue layers [16], which supports the absolute movement observed at t2.

Furthermore, IAMT is granted to affect the receptors located extensively in the fascial tissue through manual stimulation [65,66,67,68,69] and the local tension regulation comparable to acupressure and trigger point therapy as well as the segmental via the central interconnection at the spinal cord [70,71]. These treatment approaches improve function in patients with myofascial pain syndrome [72], are more successful than muscle relaxants for chronic headaches [73], and positively affect the ROM and systemic inflammatory responses after sport [74]. Moreover, the intensive mechanical stimulation at the pressure pain threshold has been assumed to exert a negative stimulation, especially to the nociceptors, with a resulting protective reaction of the organism [75], which would explain the unexpected short-term effect observed after IAMT.

Herein, the significant decrease in the SM between the SF and TLF-SL after IAMT compared with that after the placebo treatment can be explained by the dominant short-term effects on the TLF-SL. The differences in the SM within the TLF between the SL and DL recorded by Langevin et al. (2011), between healthy individuals and patients with chronic LBP and the TLF mobility determined cumulatively of the SL and DL by Griefahn et al. (2017) after a single foam roller intervention, were not confirmed in the present study [30,33]. This may be because Griefahn et al. (2017) used large-scale applications throughout the myofascial chain [33]. Both only analysed the SM and did not compare the changes in the absolute movement of the two tissue layers. Therefore, the lack of intervention effect on the SM of the TLF in this pilot study could be attributed to the systematic response after IAMT on the absolute movement of both tissue layers because it was impossible to assess whether one of them was more affected by a foam roller intervention.

The significantly improved PSLR after IAMT in this pilot study confirms the positive effects of IAMT on the functionality and ROM found in previous studies [3,4,22]. Laudner et al. (2014) demonstrated these effects on passive horizontal adduction and internal rotation of the shoulder after a single 40 s treatment compared with a classic non-treatment comparison group [4]. IAMT of the hamstrings and quadriceps lasting only 2 min approximately doubled the percentage increase in the movement of the knee and hip joint compared with an identically timed foam roller intervention. The measured gain in movement was still present to an attenuated degree 24 h after the intervention [3]. Moon et al. (2017) demonstrated a therapeutic interaction of the known connection between hamstring flexibility [76] and tightness [77,78] and the lower back [22] in patients with nonspecific LBP. A single 60 s IAMT of the hamstrings improved the hamstring flexibility and significantly reduced LBP compared with static stretching [22].

The delayed difference in the PSLR between the IG and CG and the measured superficial skin temperature suggest that the functional properties are also influenced by more than merely internal or external tissue warming. As in comparable studies with heat applications of 50–60 °C [42,55,56,57], the CG demonstrated a sharp increase in temperature [13] and improved flexibility [79], followed by a rapid return of the transient hyperaemia [40,42] to baseline levels within 30–45 min depending on the application time (10–20 min) [40,42]. Nevertheless, IAMT yielded a longer-lasting temperature increase and resulting hyperaemia accompanied by a lasting effect on hamstring flexibility, which is likely attributed to the expected change in viscosity [61] affecting the corresponding myofascial chain [23,25,26,27,79,80]. However, the strong increase in the ROM after IAMT suggests that, similar to the structural properties, other effects apart from hyperaemia (e.g., mechanical and sensory impacts) influence flexibility.

The outcomes of the superficial skin temperature in all groups led to the cautious hypothesis that a globally increased blood redistribution occurs owing to the mechanical effects. Thereby, the radiated influence on the superficial skin temperature of the contralateral side appears to be too small to affect the structural or functional properties. The clear drop in the superficial skin temperature immediately after the placebo treatment is likely due to the room-temperature placebo ultrasound application and seems to completely normalise at t2 based on the spatial and examination-specific conditions (room temperature and clothing removed), which is supported by the reference points of the PG and CG. The observed swelling at the SF on ultrasound after IAMT could indicate the expected microtraumas on the structural level and, subsequently, the discussed enrichment of cytokines in the ground substance, particularly inflammatory mediators and growth factors [5]. However, further systematic investigations for verification are required.

### Strengths and Limitations

This pilot study had a randomised, controlled design that utilised blinding and a clear standardisation of the examination and treatment protocol. To the best of our knowledge, the carefully selected multiplicity of analyses to complement the previously utilised functional movement parameters with promising but previously non-existent structural insights into the gliding motion of individual tissue layers does not appear to negatively influence the results. However, the filigree structures and the lack of collagenous (white) tissue in the muscle posed a challenge for the CCS in some cases and may explain the variance in the values, especially for the CG. A clearer delineation may be possible using a higher-resolution ultrasound transducer. We recommend that in future studies, the contact gel and, for equal baseline situations, the Fazer tool be warmed to body temperature. Whether more significant changes are seen in larger and broader collectives–men, other kinds of sports, patients with LBP, or athletes with recurrent hamstring injuries–can only be speculated at this point. Upcoming studies including an additional later post-interventional control measurement (e.g., a few hours or 24 h later, according to Markovic (2015) [3]) and a more detailed examination of the microcirculation (e.g., use of the Oxygen to See (O2C) device (LEA Medizintechnik, Heuchelheim, Germany) are recommended [81,82].

## 5. Conclusions

With its vigorous mechanical stimulation, IAMT yielded a short-term reduction in the absolute movement of the TLF-SL, immediately followed by a stronger improvement, while a slight cold stimulus led to a short-term improvement. Based on the dispositive influence of the TLF-SL, an additional decrease in the SM to the SF was recorded immediately after the intervention. Accordingly, IAMT of the lower back positively influenced hamstring flexibility, supporting the initial hypothesis of an inverse interaction, presumably via the myofascial chains through the TLF as a mechanical pivot and an anchor point of the back side of the body [23,24,25,26,27]. However, on the structural and functional levels, the short-term effects within the IG and PG were inconsistent, whereas, at t2, the same expected trends were observed. The concrete mechanisms behind these findings remain unclear. Therefore, future studies should investigate the discussed mechanical and sensory impacts, taking larger populations and additional target groups into account.

## Figures and Tables

**Table 1:** Quantitative and qualitative baseline characteristics.

Parameter		All Groups (*n* = 67)			IG (*n* = 23)			CG (*n* = 22)			PG (*n* = 22)			*p*-Value	
		Mean/*n*	±	SD	Mean/*n*	±	SD	Mean/*n*	±	SD	Mean/*n*	±	SD		
Age [year]		20.9	±	3.9	21.2	±	4.1	20.9	±	3.6	20.8	±	4.1	0.945	^‡^
Height [cm]		167.4	±	5.5	166.9	±	4.3	168.3	±	5.7	167.1	±	6.4	0.563	^‡^
Weight [kg]		62.6	±	7.0	62.7	±	8.1	62.8	±	6.4	62.4	±	6.7	0.840	^‡^
BMI [kg/m²]		22.3	±	2.3	22.5	±	2.6	22.1	±	1.7	22.4	±	2.4	0.872	^†^
Sport experience [year]		12.4	±	4.2	12.4	±	3.6	11.6	±	4.5	13.1	±	4.7	0.488	^†^
Menstruation [day]		16.4	±	8.2	16.6	±	8.6	16.1	±	8.2	16.4	±	8.2	0.963	^‡^
Weekly extent [hour]		7.2	±	2.0	7.1	±	1.9	6.7	±	1.9	7.8	±	2.3	0.222	^‡^
7-day-extent [hour]		5.1	±	3.1	4.7	±	3.0	5.1	±	3.0	5.7	±	3.4	0.536	^†^
Flexibility [0–10]		2.88	±	2.19	2.64	±	2.00	3.23	±	2.63	2.78	±	1.94	0.807	^‡^
Pain [0–10]		1.66	±	2.03	1.66	±	1.88	2.17	±	2.65	1.13	±	1.32	0.806	^‡^
Level	State	24			7			10			7				
	National	41			15			12			14			0.722	^#^
	International	2			1			0			1				
Squad	None	50			16			18			16			0.875	^#^
	State	7			3			2			2				
	National	10			4			2			4				
Free leg	Right	56			19			18			19			0.909	^#^
	Left	11			4			4			3				
MSR	Yes	52			18			16			18			0.767	^#^
	No	15			5			6			4				
Hormonal	Yes	26			9			8			9			0.953	^#^
contraception	No	41			14			14			13				

Quantitative data are presented as mean values ±SD and qualitative data as numbers (*n*). Menstruation: All: *n* = 61; IG: *n* = 21; CG: *n* = 19; PG: *n* = 21; Test: † analysis of variance, ‡ Kruskal–Wallis, # chi-squared. IG: intervention group; CG: comparison group; PG: placebo group; SD: standard deviation; BMI: body mass index; weekly extent: scheduled weekly training time; 7-day-extent: training time in the last 7 days; flexibility: subjectively perceived flexibility immediately before baseline measurement; pain: state of the lower back immediately before baseline measurement; free leg: playing/striking leg; MSR: myofascial self-release experience.

**Table 2:** Structural Movement Parameters.

Parameter	IG (*n* = 23)						CG (*n* = 22)						PG (*n* = 22)						*p*-Value	
	Mean	±	SD	Δ1/Δ2	±	SD	Mean	±	SD	Δ1/Δ2	±	SD	Mean	±	SD	Δ1/Δ2	±	SD		
**Baseline (t0)**																				
SF R [mm]	3.52	±	1.59				3.73	±	1.59				3.40	±	1.88				0.808	^†^
TLF-SL R [mm]	5.85	±	2.45				5.83	±	2.60				4.73	±	2.72				0.265	^†^
TLF-DL R [mm]	10.58	±	3.80				11.14	±	3.92				9.21	±	4.01				0.249	^†^
ESM R [mm]	14.07	±	3.17				16.63	±	3.14				14.03	±	3.78				**0.017**	^*†^
SF L [mm]	3.52	±	1.54				3.88	±	1.47				3.34	±	1.36				0.467	^†^
TLF-SL L [mm]	6.82	±	2.75				7.10	±	3.30				5.74	±	2.34				0.248	^†^
TLF-DL L [mm]	10.36	±	2.88				11.51	±	3.88				9.79	±	2.80				0.202	^†^
ESM L [mm]	13.77	±	2.69				14.22	±	3.12				13.27	±	3.54				0.604	^†^
SM (SF/TLF-SL) R [mm]	2.33	±	1.30				2.10	±	1.46				1.34	±	1.06				**0.031**	^*†^
SM (TLF-SL/TLF-DL) R [mm]	4.73	±	2.98				5.31	±	2.51				4.48	±	2.69				0.449	^‡^
SM (TLF-DL/ESM) R [mm]	3.50	±	3.27				5.49	±	3.33				4.83	±	3.66				0.144	^†^
SM (SF/TLF-SL) L [mm]	3.30	±	1.86				3.22	±	2.08				2.40	±	1.29				0.183	^†^
SM (TLF-SL/TLF-DL) L [mm]	3.54	±	1.75				4.41	±	2.28				4.05	±	2.10				0.364	^†^
SM (TLF-DL/ESM) L [mm]	3.41	±	3.31				2.71	±	3.61				3.48	±	3.07				0.697	^†^
**1st Post (t1)**																				
SF R [mm]	3.17	±	1.87	−0.35	±	1.66	3.94	±	1.82	0.21	±	1.72	3.73	±	1.79	0.33	±	1.33	0.307	^†^
TLF-SL R [mm]	5.19	±	2.99	−0.66	±	2.33	5.65	±	2.52	−0.18	±	2.16	5.73	±	2.90	1.00	±	1.95	**0.036**	^*†^
TLF-DL R [mm]	10.15	±	3.83	−0.43	±	3.72	11.00	±	3.69	−0.14	±	2.27	9.95	±	3.19	0.74	±	2.75	0.402	^†^
ESM R [mm]	13.65	±	3.84	−0.43	±	2.84	15.28	±	4.12	−1.35	±	3.17	14.42	±	3.61	0.38	±	3.02	0.170	^†^
SF L [mm]	3.33	±	1.54	−0.19	±	1.34	4.13	±	1.86	0.25	±	1.15	3.37	±	1.50	0.03	±	0.65	0.416	^†^
TLF-SL L [mm]	6.64	±	2.70	−0.18	±	1.79	7.25	±	3.72	0.15	±	1.72	5.80	±	2.39	0.05	±	1.03	0.770	^†^
TLF-DL L [mm]	10.71	±	2.75	0.35	±	1.82	11.64	±	4.52	0.12	±	2.26	9.47	±	2.71	−0.32	±	1.50	0.485	^†^
ESM L [mm]	14.00	±	3.53	0.24	±	2.93	14.66	±	3.33	0.44	±	2.59	13.53	±	3.40	0.26	±	2.57	0.962	^†^
SM (SF/TLF-SL) R [mm]	2.02	±	1.51	−0.31	±	1.32	1.71	±	1.04	−0.39	±	0.92	2.01	±	1.34	0.67	±	1.13	**0.004**	^*†^
SM (TLF-SL/TLF-DL) R [mm]	4.96	±	2.40	0.23	±	2.10	5.35	±	3.01	0.04	±	2.00	4.22	±	2.41	−0.26	±	1.82	0.464	^‡^
SM (TLF-DL/ESM) R [mm]	3.50	±	3.36	0.00	±	2.87	4.28	±	3.82	−1.21	±	2.58	4.47	±	2.96	−0.36	±	2.47	0.299	^†^
SM (SF/TLF-SL) L [mm]	3.31	±	1.79	0.01	±	1.21	3.13	±	2.10	−0.10	±	0.90	2.43	±	1.21	0.03	±	0.78	0.902	^†^
SM (TLF-SL/TLF-DL) L [mm]	4.06	±	2.01	0.52	±	1.41	4.38	±	1.97	−0.03	±	1.28	3.67	±	1.61	−0.38	±	1.20	0.072	^†^
SM (TLF-DL/ESM) L [mm]	3.30	±	3.51	−0.11	±	2.28	3.03	±	3.58	0.32	±	2.11	4.06	±	2.89	0.58	±	2.09	0.556	^†^
**2nd Post (t2)**																				
SF R [mm]	4.04	±	1.72	0.52	±	1.94	4.02	±	1.92	0.30	±	1.68	3.41	±	1.60	0.01	±	1.11	0.581	^†^
TLF-SL R [mm]	6.12	±	2.33	0.27	±	2.31	6.04	±	3.01	0.21	±	2.12	5.13	±	2.34	0.40	±	1.68	0.951	^†^
TLF-DL R [mm]	10.93	±	3.74	0.35	±	3.42	11.61	±	4.27	0.47	±	2.67	9.29	±	3.64	0.08	±	3.22	0.913	^†^
ESM R [mm]	14.70	±	3.45	0.63	±	2.96	15.80	±	4.02	−0.84	±	2.91	14.34	±	2.87	0.31	±	3.04	0.230	^†^
SF L [mm]	3.58	±	1.79	0.06	±	1.46	4.13	±	1.43	0.25	±	1.01	3.08	±	1.41	−0.27	±	0.93	0.336	^†^
TLF-SL L [mm]	6.84	±	3.21	0.03	±	2.12	6.83	±	2.92	−0.27	±	1.64	5.50	±	2.57	−0.25	±	1.32	0.816	^†^
TLF-DL L [mm]	10.56	±	3.01	0.20	±	2.03	11.59	±	4.32	0.08	±	2.26	8.98	±	3.01	−0.81	±	1.92	0.217	^†^
ESM L [mm]	13.31	±	3.23	−0.46	±	3.15	14.99	±	3.94	0.77	±	1.85	13.22	±	3.27	−0.05	±	2.10	0.240	^†^
SM (SF/TLF-SL) R [mm]	2.08	±	1.39	−0.25	±	1.24	2.02	±	1.47	−0.09	±	1.10	1.72	±	1.08	0.39	±	1.08	0.162	^†^
SM (TLF-SL/TLF-DL) R [mm]	4.81	±	2.58	0.08	±	1.95	5.57	±	3.12	0.27	±	1.79	4.16	±	2.94	−0.32	±	1.96	0.619	^‡^
SM (TLF-DL/ESM) R [mm]	3.78	±	3.08	0.28	±	2.66	4.18	±	3.10	−1.31	±	2.18	5.06	±	3.69	0.23	±	3.87	0.139	^†^
SM (SF/TLF-SL) L [mm]	3.26	±	1.88	−0.03	±	0.97	2.70	±	1.74	−0.52	±	0.94	2.42	±	1.54	0.02	±	0.95	0.122	^†^
SM (TLF-SL/TLF-DL) L [mm]	3.72	±	1.98	0.18	±	1.50	4.76	±	2.70	0.35	±	1.48	3.48	±	1.88	−0.56	±	1.74	0.131	^†^
SM (TLF-DL/ESM) L [mm]	2.75	±	2.51	−0.66	±	2.44	3.40	±	3.22	0.69	±	2.13	4.24	±	2.65	0.76	±	1.66	**0.044**	^*†^

Mean values, Δ1 (t1 − t0) and Δ2 (t2 − t0) ± SD of the absolute movement and shear motion are shown. * *p* < 0.05. Test: † analysis of variance, ‡ Kruskal–Wallis. IG: intervention group; CG: comparison group; PG: placebo group; SD: standard deviation; t0: before the intervention; t1: immediately after the intervention; t2: 45 min after the intervention; SF: superficial fascia; TLF: thoracolumbar fascia; SL: superficial lamina; DL: deep lamina; ESM: erector spinae muscle; R: right; L: left; SM: shear motion.

**Table 3:** Functional Movement Parameters.

Parameter	IG (*n* = 23)						CG (*n* = 22)						PG (*n* = 22)						*p*-Value	
	Mean	±	SD	Δ1/Δ2	±	SD	Mean	±	SD	Δ1/Δ2	±	SD	Mean	±	SD	Δ1/Δ2	±	SD		
**Baseline (t0)**																				
PSLR R [°]	77.4	±	10.9				72.6	±	11.3				77.6	±	15.0				0.337	^†^
PSLR L [°]	77.2	±	13.0				72.6	±	10.6				76.6	±	14.2				0.430	^†^
PLS [°]	52.7	±	8.2				53.6	±	9.6				54.9	±	9.5				0.717	^†^
ALS [°]	54.3	±	9.0				54.7	±	9.3				56.0	±	10.0				0.708	^‡^
PTS [°]	29.3	±	9.5				27.7	±	12.4				25.6	±	8.2				0.467	^†^
ATS [°]	31.6	±	10.5				28.3	±	10.2				25.7	±	9.2				0.180	^‡^
**1st Post (t1)**																				
PSLR R [°]	79.3	±	10.8	1.9	±	3.6	73.0	±	10.3	0.4	±	2.1	77.4	±	14.8	−0.2	±	2.5	**0.044**	^*†^
PSLR L [°]	77.5	±	12.7	0.3	±	2.4	72.8	±	9.4	0.2	±	2.4	77.2	±	13.5	0.6	±	2.2	0.865	^†^
PLS [°]	51.7	±	8.3	−1.0	±	4.9	52.9	±	6.9	−0.7	±	4.2	55.2	±	8.0	0.3	±	4.1	0.616	^†^
ALS [°]	53.3	±	8.4	−1.0	±	4.2	53.8	±	8.4	−0.9	±	4.3	55.7	±	7.9	−0.3	±	4.5	0.776	^‡^
PTS [°]	30.5	±	8.9	1.2	±	7.0	30.9	±	9.5	3.2	±	8.1	25.4	±	9.7	−0.2	±	6.9	0.319	^†^
ATS [°]	32.4	±	7.9	0.8	±	5.7	31.2	±	10.0	2.9	±	6.1	27.8	±	10.3	2.1	±	5.0	0.523	^‡^
**2nd Post (t2)**																				
PSLR R [°]	79.7	±	9.1	2.3	±	3.6	72.9	±	11.4	0.3	±	2.3	77.0	±	14.9	−0.6	±	1.8	**0.002**	^*†^
PSLR L [°]	77.5	±	12.0	0.3	±	2.7	73.0	±	10.3	0.4	±	2.9	76.6	±	14.4	−0.1	±	2.4	0.838	^†^
PLS [°]	50.7	±	11.2	−2.0	±	7.6	52.9	±	9.3	−0.7	±	3.9	54.8	±	7.4	−0.1	±	5.3	0.537	^†^
ALS [°]	52.1	±	13.6	−2.2	±	12.7	53.3	±	9.2	−1.5	±	5.1	55.7	±	8.1	−0.3	±	6.0	0.654	^‡^
PTS [°]	28.7	±	8.5	−0.6	±	7.5	30.6	±	10.2	2.8	±	9.2	27.0	±	11.1	1.5	±	7.6	0.367	^†^
ATS [°]	31.0	±	8.6	−0.6	±	5.6	30.5	±	9.5	2.2	±	5.9	27.0	±	11.9	1.3	±	5.7	0.415	^‡^

Mean values, Δ1 (t1 − t0) and Δ2 (t2 − t0) ± SD of the passive straight leg raise test (PSLR) and double inclinometry are shown. * *p* < 0.05. Test: † analysis of variance, ‡ Kruskal–Wallis. IG: intervention group; CG: comparison group; PG: placebo group; SD: standard deviation; t0: before the intervention; t1: immediately after the intervention; t2: 45 min after the intervention; PLS: passive lumbar spine; ALS: active lumbar spine; PTS: passive thoracic spine; ATS: active thoracic spine; R: right; L: left; °: degree.

**Table 4:** Superficial skin temperature.

Parameter	IG (*n* = 23)						CG (*n* = 22)						PG (*n* = 22)						*p*-Value	
	Mean	±	SD	Δ1/Δ2	±	SD	Mean	±	SD	Δ1/Δ2	±	SD	Mean	±	SD	Δ1/Δ2	±	SD		
**Baseline (t0)**																				
Temp LS R [°C]	33.4	±	0.8				33.5	±	0.6				33.4	±	0.8				0.966	^‡^
Temp LS L [°C]	33.5	±	0.8				33.7	±	0.7				33.5	±	0.9				0.941	^‡^
Temp Ref [°C]	29.9	±	1.0				30.4	±	1.1				30.2	±	1.0				0.358	^‡^
**1st Post (t1)**																				
Temp LS R [°C]	34.9	±	0.6	1.5	±	0.5	35.7	±	0.3	2.2	±	0.6	31.4	±	1.1	−2.0	±	0.7	**0.001**	^*‡^
Temp LS L [°C]	33.7	±	0.9	0.2	±	0.5	34.1	±	0.7	0.4	±	0.4	32.9	±	0.9	−0.6	±	0.6	**0.001**	^*‡^
Temp Ref [°C]	30.0	±	1.0	0.1	±	0.8	30.3	±	1.0	−0.1	±	0.6	30.1	±	1.1	−0.1	±	0.8	0.886	^‡^
**2nd Post (t2)**																				
Temp LS R [°C]	34.0	±	0.5	0.7	±	0.6	33.8	±	0.5	0.3	±	0.5	33.0	±	0.7	−0.4	±	0.4	**0.001**	^*‡^
Temp LS L [°C]	33.6	±	0.7	0.1	±	0.4	33.6	±	0.7	−0.1	±	0.5	33.3	±	0.7	−0.2	±	0.6	0.159	^‡^
Temp Ref [°C]	29.8	±	0.8	−0.1	±	1.0	30.0	±	0.8	−0.4	±	0.7	29.9	±	0.9	−0.3	±	0.9	0.561	^‡^

Mean values, Δ1 (t1 − t0) and Δ2 (t2 − t0) ± SD of the superficial skin temperature are shown. * *p* < 0.05. Test: ‡ Kruskal–Wallis. IG: intervention group; CG: comparison group; PG: placebo group; SD: standard deviation; t0: before the intervention; t1: immediately after the intervention; t2: 45 min after the intervention; Temp: superficial skin temperature; LS: lumbar spine; Ref: reference point; R: right; L: left.

## Data Availability

The data presented in this study are available in the text, figures, and tables.
